# Dobutamine stress test and beta-agonist - a potential concern for nuclear cardiology testing: a case report

**DOI:** 10.4076/1757-1626-2-7466

**Published:** 2009-07-09

**Authors:** Cevher Ozcan, Barry L Zaret

**Affiliations:** Department of Medicine, Section of Cardiovascular Medicine, Yale University School of Medicine333 Cedar Street, 3FMP New Haven, Connecticut, 06520USA

## Abstract

**Introduction:**

Chest pain with ST-segment elevation is a rare clinical problem during dobutamine stress testing. Although beta-agonists treatment prior to dobutamine stress testing has been shown to reduce the duration and amount of dobutamine infusion and atropine requirement, there is insufficient information about potential complications of this pharmacologic combination.

**Case presentation:**

We present a 67-year-old patient with dobutamine stress testing -induced chest pain and ST elevation who received albuterol for clinical treatment of bronchospastic disease prior to the test. She developed persistent chest pain and ST elevation despite medical management. Urgent cardiac catheterization showed no significant obstructive coronary artery disease. Thus coronary artery spasm was likely responsible for the chest pain and electrocardiogram abnormality in our patient as a result of β-agonist and dobutamine combination.

**Conclusions:**

Beta-agonists pre-treatment with dobutamine stress testing may induce coronary spasm in association with chest pain and ST elevation. Clinicians and nuclear cardiologist should be aware of this potential side effect of β-agonists treatment with dobutamine stress testing, particularly since dobutamine stress testing in nuclear cardiology is done in patient with chronic obstructive lung disease.

## Introduction

Dobutamine infusion in combination with radionuclide myocardial perfusion imaging is an alternative pharmacological stress test for detection of myocardial ischemia in patient who is not able to tolerate dipyridamole or adenosine infusion. It is commonly used in patients with chronic obstructive pulmonary disease (COPD). As a catecholamine with predominant β1 receptor against, dobutamine increases heart rate, systolic blood pressure and myocardial contractility. Consequently, myocardial oxygen demand increases. Although its hemodynamic effect is similar to physical exercise, dobutamine stress test (DST) is not a substitute for exercise stress test. Serious side effects may occur during DST including myocardial infarction, ventricular arrhythmia, hypotension and prolonged ischemia [1-3]. However ST-segment elevation (STSE) with chest pain is a rare event in DST with myocardial perfusion imaging.

Numerous studies have demonstrated that DST in association with radionuclide or echocardiographic imaging is sensitive and specific diagnostic test for coronary artery disease. In order to maintain sensitivity of DST, it is important to reach target heart rate during the test. Atropine is usually combined with dobutamine infusion to increase heart rate. It has been demonstrated that β-agonists treatment shortly prior to DST reduced amounts and duration of dobutamine infusion as well as requirement of atropine [4]. However it is not clear whether the incidence of ST-segment elevation with chest pain increases with the β-agonist and dobutamine combination. This is particularly important for the patients who undergo DST with nuclear imaging since the number of studies is limited and this patient population has multiple co-morbidities.

## Case presentation

**Figure 1.**
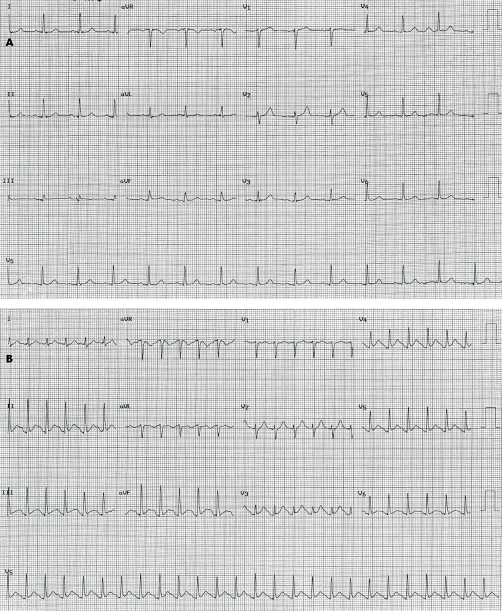
Electrocardiogram at baseline **(A)** and recovery phase of dobutamine stress test in association with chest pain **(B)**, which shows ST-segment elevation in lead II, III, aVF, V3, V4 and V5.

**Figure 2.**
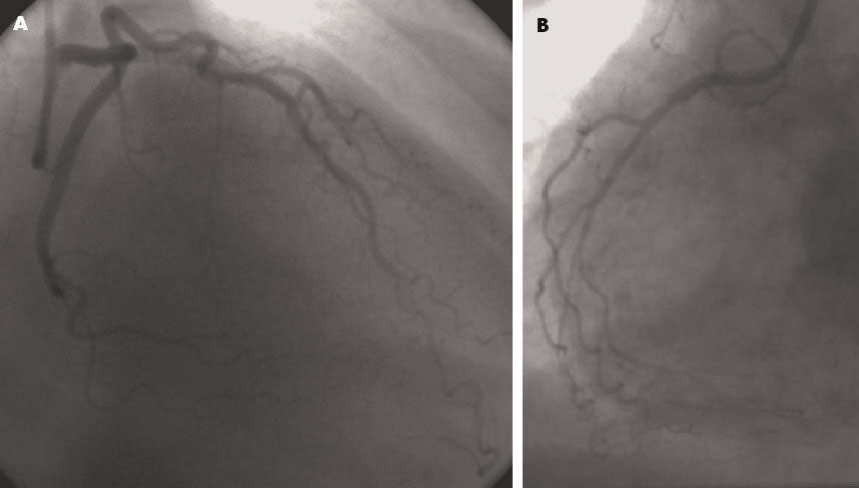
Coronary angiograms demonstrate no obstructive disease at left **(A)** and right **(B)** coronary artery systems.

A 67-year-old Caucasian female with severe COPD who was receiving continuous oxygen treatment in addition to multiple bronchodilator and ant-inflammatory medications including fluticasone/salmeterol (Advair), tiotropium-bromide (Spiriva), ipratropium-bromide/albuterol-sulfate (DuoNeb) and budesonide (Pulmicort), presented to the hospital with sudden onset substernal chest pressure at rest which resolved spontaneously. She has no history of coronary artery disease, but limited cardiovascular risk factors including age and hypertension. Her physical examination was unremarkable with the exception of elevated blood pressure (158/92 mmHg). Serial electrocardiogram and cardiac enzymes during hospital course were within normal limits. For that reason, DST was scheduled. The patient received albuterol inhaler treatment 30 minutes prior to DST in addition to her scheduled bronchodilators as above. She underwent pharmacological stress test with dobutamine infusion at a maximum rate of 40 mcg/kg/min, a total dose of 28 mg, and atropine 0.2 mg. She reached to target heart rate with dobutamine/atropine combination. Blood pressure response was normal. During recovery the patient developed severe substernal chest pain that was similar to her prior pain and radiated to jaw. She appeared diaphoretic, pale and sick. Concurrent electrocardiogram showed 1.5 mm STSE in lead II, III, aVF, V3, V4 and V5 (Figure 1A & B). There was only minimal improvement of chest pain with sublingual nitroglycerine. ST-segment remained elevated. Thus the patient underwent urgent cardiac catheterization. Coronary angiogram revealed no obstructive epicardial coronary artery disease (Figure 2A & B). Chest pain resolved during catheterization while she was receiving nitroglycerine infusion and morphine. Thus our patient likely manifested coronary spasm with chest pain and STSE in the setting of albuterol and dobutamine combination. There was no evidence of myocardial necrosis based on biomarkers. The patient’s hospital course was uncomplicated. She remained chest pain free with medical management including long acting nitroglycerine.

## Discussion

Pre-treatment with β-agonists in DST reduces the time of the test and the use of atropine in addition to requiring lesser amount of dobutamine infusion. However it is not known whether β-agonists may have additive effect on inducing coronary spasm and related STSE with angina during DST. Physiologic response to dobutamine and β-agonists on circulatory system are similar that may enhance overall effects of each other.

STSE during stress test occurs in the presence of myocardial ischemia, coronary spasm or left ventricular dysfunction. It is rare clinical problem in patients without previous infract or coronary artery disease and associated with coronary spasm or non-occlusive coronary lesion [2,5]. Typically STSE secondary to coronary spasm occurs in recovery phase of DST with no response to β-blocker [2,4-6].

The mechanism of dobutamine induced vasospasm with or without β-agonists is complex. Dobutamine primarily stimulates cardiomyocyte sarcolemma β1-adrenergic receptors, but also has effect on β2- and α1-receptors. Increase in myocardial oxygen demand and coronary flow causes α-adrenergic mediated vasoconstriction that has been observed during exercise. In fact exercise-induced coronary spasm is a result of α1 receptor stimulation. Higher doses of dobutamine, as in DST, generate more α1-adrenergic effect that may induce coronary spasm, particularly in the presence of endothelial dysfunction [6]. Moreover, dobutamine-induced coronary vasoconstriction is mediated through postjunctional α2-adrenergic receptors [7].

β-agonists cause cardiovascular side effects including angina, tachyarrhythmias, hypertension and myocardial injury by increasing myocardial oxygen consumption. Albuterol activates β2-adrenergic receptors particularly in coronary resistant vessels that are partially mediated by the endothelium at the microcirculatory level. Endothelial function plays important role in β2-agonist effect on vascular tone. Intracoronary infusion of salbutamol induces vasoconstriction in stenotic coronary arteries without increase in heart rate and blood pressure [8]. Underlying endothelial dysfunction was likely a reason for our patient to develop coronary spasm in response to albuterol and dobutamine combination since she had no obstructive coronary disease.

## Conclusions

Albuterol pre-treatment with DST may induce coronary spasm in association with chest pain and STSE. This clinical condition may lead to invasive procedures like cardiac catheterization and possible complications. Although the true incidence of coronary vasospasm with STSE during DST with albuterol pre-treatment is not known, nuclear cardiologist must be aware of this potentially life threatening complication of this non-invasive diagnostic test. Coronary spasm should be considered in all patients with STSE and chest pain during DST, particularly following β-agonist treatment. The safety of this combination must be re-examined. Thus, the patients should not be subjected to the DST if they are treated with β-agonist before the test.

## Abbreviations

COPD: Chronic obstructive pulmonary disease
DST: Dobutamine stress test
STSE: ST-segment elevation

## References

[bib-001] Picano E, Mathias W, Pingitore A, Bigi R, Previtali M (1994). Safety and tolerability of dobutamin-atropine stress echocardiography: A prospective, multicenter study. Lancet.

[bib-002] Elhendy A, Geleijnse ML, Roelandt JR, van Domburg RT, Cornel JH, TenCate FJ, Postma-Tjoa J, Reijs AE, el-Said GM, Fioretti PM (1995). Evaluation by quantitative 99m-technetium MIBI SPECT and echocardiography of myocardial perfusion and wall motion abnormalities in patients with dobutamine-indiced ST-segment elevation. Am J Cardiol.

[bib-003] Lubitz SA, Duvall WL, Kim MC, Henzlova MJ (2007). Dobutamin-induced myocardial infarction with normal coronary arteries during stress SPECT myocardial perfusion imaging. J Nucl Cardiol.

[bib-004] Desai MY, De la Pena-Almaguer E, Mannting F (2001). Can pre-treatment with B-agonists reduce stress test time and the use of atropine in dobutamine stress testing. Cardiology.

[bib-005] Previtali M, Fetiveau R, Lanzarini L, Cavalotti C (1998). Dobutamine-induced ST-segment elevation in patients without myocardial infarction. Am J Cardiol.

[bib-006] Kawano H, Fujii H, Motoyama T, Kugiyama K, Ogawa H, Yasue H (2000). Myocardial ischemia due to coronary artery spasm during dobutamin stress echocardiography. Am J Cardiol.

[bib-007] Dai XZ, Chen DG, Bache RJ (1989). Alpha-adrenergic effects of dopamine and dobutamine on the coronary circulation. J Cardiovasc Pharmacol.

[bib-008] Barbato E, Piscione F, Bartunek J, Galasso G, Cirillo P, De Luca G, Iaccarino G, De Bruyne B, Chiariello M, Wijns W (2005). Role of B2 adrenergic receptors in human atherosclerotic coronary arteries. Circulation.

